# Cross-border rare disease advocacy: Preethi Krishnaraj interviews Harsha Rajasimha

**DOI:** 10.1242/dmm.050672

**Published:** 2024-01-25

**Authors:** Preethi Krishnaraj, Harsha K. Rajasimha

**Affiliations:** ^1^ Max Planck Institute for Heart and Lung Research, Department of Developmental Genetics, 61231 Bad Nauheim, Germany; ^2^ Indo US Organization for Rare Diseases (IndoUSrare), Herndon, VA, USA; ^3^ Jeeva Clinical Trials, Inc., Manassas, VA, USA; ^4^ Affiliate Faculty, School of Systems Biology, George Mason University, Fairfax, VA, USA

Rare diseases affect only a few people in a given population but, collectively, they impact over 400 million people globally (Nguengang Wakap et al., 2020). It is estimated that there are more than 10,800 rare diseases (RareX), 72% of which have genetic origin (Nguengang Wakap et al., 2020). Diagnosis of rare diseases, encompassing congenital disorders, metabolic disorders, autoimmune disorders and certain types of cancer, often takes 5 to 7 years and, in ultra-rare cases, a diagnosis may never be achieved (Haendel et al., 2020). Unfortunately, 95% of rare diseases do not have recognized treatments (Global Genes). These diseases are also referred to as orphan diseases, as they are often neglected by medical and research endeavors.

**Figure:**
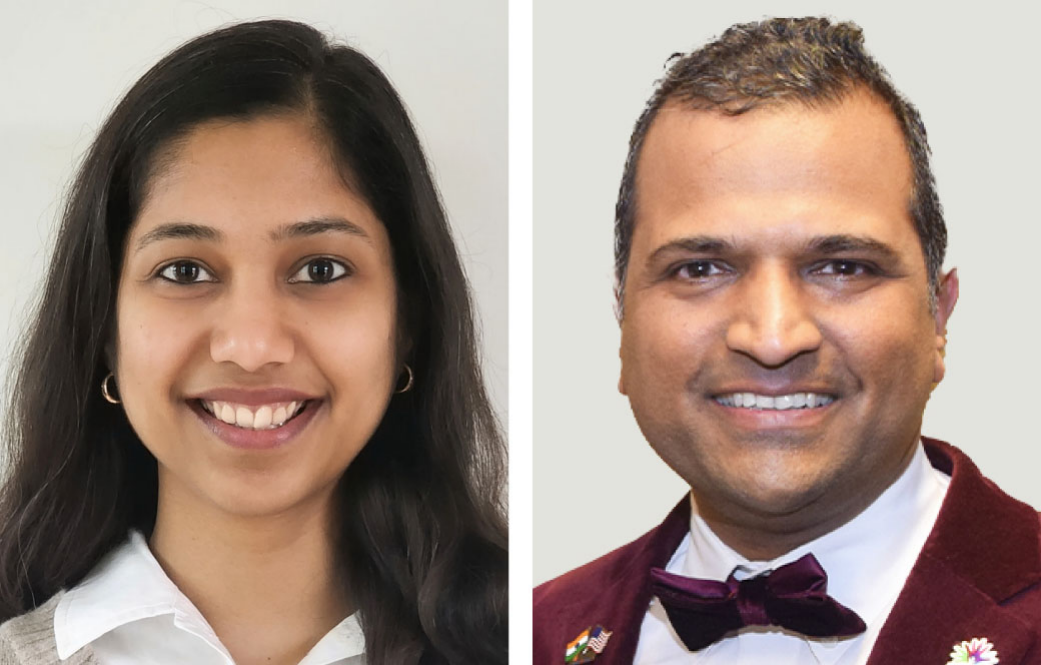
Preethi Krishnaraj (left) and Harsha Rajasimha (right)

**Figure:**
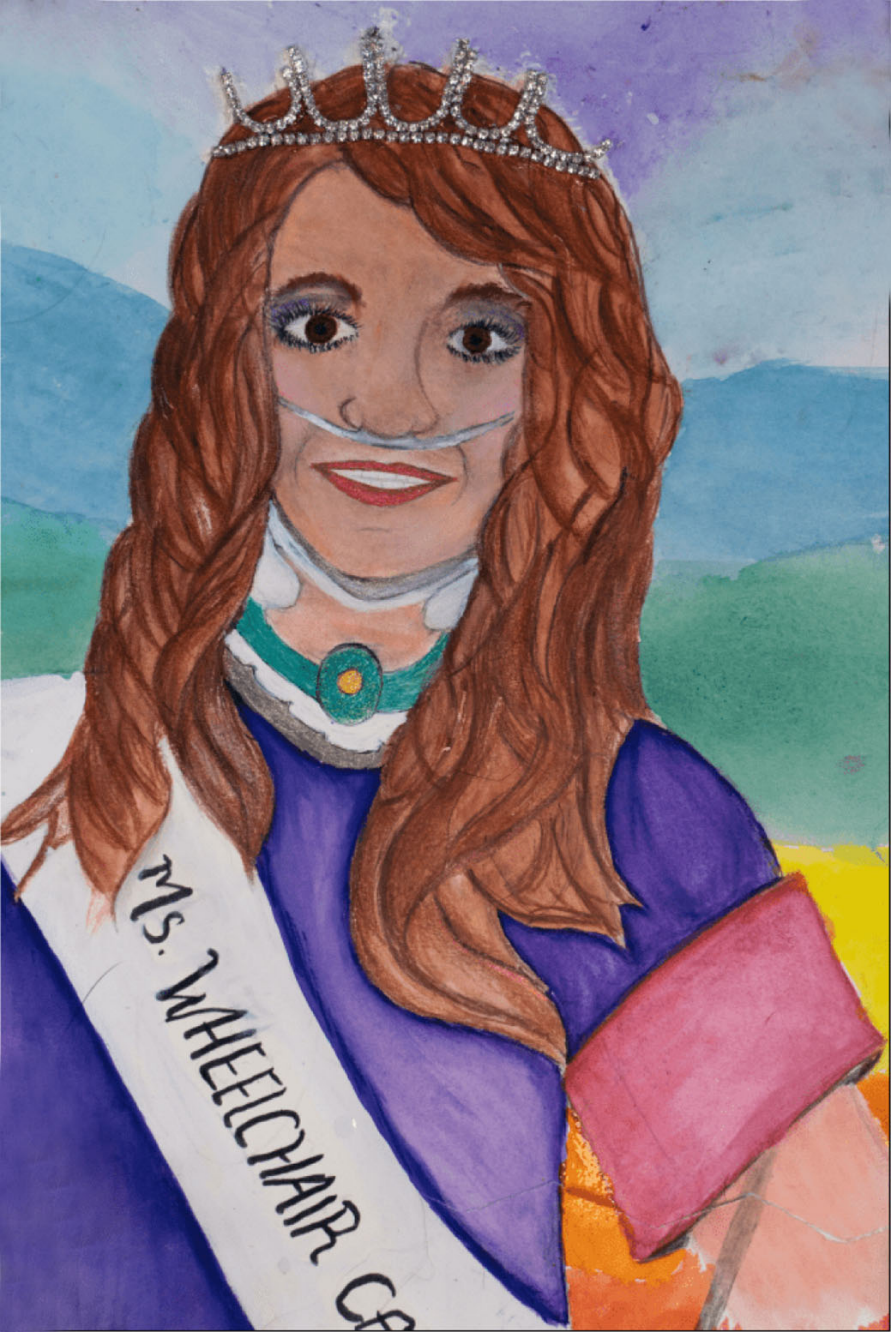
**‘Living rare with confidence’.** A self-portrait of Ms Wheelchair California 2022, Deborah Vick, for ART4RARE, a global rare-disease day art competition, organized by IndoUSrare. This image is courtesy of IndoUSrare. Copyright 2024. All rights reserved. This image is not published under the CC-BY license of the article.

Patient advocacy groups are instrumental in addressing the unique challenges faced by people with rare diseases. Harsha Rajasimha – who did his schooling and college in Bengaluru, India – is one such rare disease advocate. In 2001 he moved to the United States for his studies and career in bioinformatics. He earned his Master's degree in Computer Science and his Doctoral degree in an interdisciplinary program – Genetics, Bioinformatics and Computational Biology, from Virginia Tech, Blacksburg, VA, USA, and later moved to the National Institute of Health (NIH), USA for his postdoctoral appointments. During this time, Harsha and his wife, endured a heart-breaking journey due to the loss of their second child to Edwards syndrome (trisomy 18). Five years later, a second crisis struck his life when his younger brother passed away due to complications from juvenile diabetes. These personal experiences motivated him to envision the Organization for Rare Diseases India (ORDI; https://ordindia.in/) (Rajasimha et al., 2014; Box 1) with six other co-founders – Prasanna Kumar Shirol, Preveen Ramamoorthy, Vijay Chandru, Madhuri Hegde, Ravinandan Eswaramurthy and Sangeeta Barde – in 2014. A few years later, in 2019, he stepped out of ORDI and founded the Indo US Organization for Rare Diseases (IndoUSrare; www.indousrare.org) (Khera et al., 2021; Box 1). Furthermore, in 2019, Harsha also founded Jeeva Clinical Trials (Jeeva; Box 1), a unified cloud-based clinical trial management software platform for sponsors of therapies for rare and common diseases to execute modern clinical research studies, patient registries or natural history studies, with efficiency and universal access. Alongside Harsha's dedication to advocacy, he held multiple leadership positions. He was a senior Director of Bioinformatics and Translational Research at Dovel Technologies, VA, USA, where he managed academic and US Federal Government projects. At George Mason University, Fairfax, VA, USA, he serves as affiliate faculty in the School of Systems Biology and leads the rare diseases research initiatives. Additionally, he worked as the global Vice President at Strand Life Sciences, a company specializing in precision medicine, specifically developing genomics-based clinical lab tests for cancer and inherited diseases. In 2016, in collaboration with the Rare Genomics Institute, Harsha was honored with Sanofi Genzyme's rare diseases Patient Advocacy Leadership award. In this conversation with Preethi Krishnaraj, Harsha shares insights into rare disease clinical trials and the importance of cross-border collaborations.

**Box 1. ORDI, IndoUSrare and Jeeva Clinical Trials**

• ORDI (Organization for Rare Diseases India) is a non-profit umbrella organization for individuals affected by rare diseases across India. The organization prioritizes rare diseases as a human rights issue, strives to minimize healthcare disparities, advocates for equitable healthcare access, public awareness and policy development. Through collaborations with national and international entities like the National Organization for Rare Disorders (NORD) in the USA, the European Organisation for Rare Diseases (EURORDIS) and Global Genes, ORDI fosters global connections, sharing knowledge and support. Additionally, ORDI actively drives research, clinical trials and orphan-drug development within the Indian rare-disease community.

• IndoUSrare (Indo US Organization for Rare Diseases) is a humanitarian non-profit 501(c)(3) tax-exempt public charity organization based in the United States. It holds events to bring these stakeholders together, with the annual Indo US Bridging RARE Summit scheduled for Nov 16-18, 2024 in New Delhi, India. Through collaborations with national and international entities – like the National Organization for Rare Disorders (NORD) in the USA, Global Genes, Undiagnosed Diseases Network International (UDNI), members of their Corporate Alliance and Patient Alliance programs – IndoUSrare fosters global connections, knowledge sharing and support. Additionally, IndoUSrare actively advocates for the inclusion of Indian Diaspora in global rare-disease research, clinical trials and orphan-drug development. The organization has achieved a platinum seal of financial transparency and a top-rated non-profit status over the years.

• Jeeva Clinical Trials is an AI-driven unified clinical trial management platform with the mission to lower the cost of drug development and accelerate the speed of clinical trials by empowering sponsors to execute modern clinical trials with a unified software platform with fewer staff members, and significantly less burden on clinical researchers and patients. Today the Jeeva eClinical platform supports sponsors and Clinical Research Organizations (CROs) across the globe, helping to reduce the logistical burdens on patients and study teams by >70%. Its complete suite of technology supports fully decentralized and hybrid clinical trials, and has resulted in being selected by research hospitals, CROs and sponsors developing pioneering therapeutics, such as Frantz Viral Therapeutics and ImmunoACT.

Preethi, originally from Chennai, India, is a PhD student from Didier Stainier's lab (Max Planck Institute for Heart and Lung Research, Bad Nauheim, Germany). During her undergraduate studies at SRM Institute of Science and Technology (formerly SRM University) in India, she met with several individuals with rare diseases while completing her genetic counselling internship. She recently connected with Harsha through his organisation IndoUSrare to gain a broader perspective of rare disease and learn how she can support the patient community. Preethi is also dedicated to raising awareness about rare disease research through science communication, as an avid edit for preLights.

Analyzing data as a scientist is very different from real-life experiences of what life looks like for a family with a member affected by a rare disease. That was the eye-opening, life-changing moment where my journey took a turn. I thought it was a call of duty to do something from my own unique experiences and perspectives for millions of people battling a rare disease.

## **Preethi:** Harsha, please share with us your journey in becoming a rare disease stakeholder. How did it all start?

**Harsha:** My journey as a rare disease stakeholder is very unusual. In 2001, I graduated from Bengaluru University, India, with a Bachelor's degree in Computer Science Engineering. It was at this time that the human genome project was completed. So, I got involved in bioinformatics, genomics and big data analysis during my Master's degree and PhD at Virginia Tech university, VA, USA. Then, I moved to the NIH and United States Food and Drug Administration (US FDA), for my early postdoctoral career in genomics and precision medicine. The Illumina sequencer was already in existence then, and only a handful of institutions in the world had access to those sequencers. I was fortunate to be part of one of them at the Neurobiology Neurodegeneration & Repair Laboratory (NNRL) at the National Eye Institute at Bethesda, MD, as the sole bioinformatician. During that phase, we analyzed genomic and multiomic data from several rare retinal disease samples, and identified mutations related to Leber's congenital amaurosis or dominant maculopathy, age-related macular degeneration, and several other retinal degenerative diseases. In 2012, like many folks in the rare disease space, I had a personal experience when my child was born with a rare disease. That was when I transitioned to be a social entrepreneur and deepened my involvement in advocacy.

Analyzing data as a scientist is very different from real-life experiences of what life looks like for a family with a member affected by a rare disease. That was the eye-opening, life-changing moment where my journey took a turn. I thought it was a call of duty to do something from my own unique experiences and perspectives for millions of people battling a rare disease.

## **Preethi:** It was then that you established ORDI and IndoUSrare?

**Harsha:** Right, I brought together a team of stakeholders across India and the US to cofound the national umbrella organization Organization for Rare Diseases India (ORDI). We published the first ever peer-reviewed article from India about the challenges and opportunities for rare diseases, highlighting the Indian scenario (Rajasimha et al., 2014). Back then, in India, there were some activities that focused on lysosomal storage diseases and specific types of rare disease, but not collectively on rare diseases. I realized there was a lot of groundwork to be done in India. For example, raising awareness and educating the general public, patients and medical doctors, providing information, facilitating connections to clinical trials focusing on rare diseases and so on. To do all these, I felt I was uniquely positioned because of my Indian-American scientist status. I experienced both cultures and their healthcare systems, and noticed significant differences and commonalities that exist between the USA and India. In the USA, the Orphan Drug Act implemented by the FDA in 1983, transformed the development of drugs for neglected and rare genetic diseases. This act motivated pharmaceutical companies and patient groups to develop new treatment options for rare diseases, and today we have over 1100 approved orphan drugs and 17 gene therapies in the US market. However, these benefits were limited to the USA and couldn't be extended to help patients in the rest of the world, including India. In addition, many countries lack an FDA-like organization, meaning they rely largely on FDA reviews for the safety and efficacy of new drugs, including orphan drugs. For instance, Indian regulators, such as the Central Drugs Standard Control Organisation (CDSCO), often accept any drug approved by the FDA for orphan use without requiring additional clinical trials, making them readily available in the Indian market. This made it evident to me that engaging the Indian diaspora – including those residing in the USA and elsewhere – is crucial. This gap inspired me to establish the Indo US organization for Rare Diseases (IndoUSrare).

## **Preethi:** Could you elaborate on how IndoUSrare facilitates drug development and cross-border collaborations?

**Harsha:** We have a Patient Alliance Program that brings together various foundations from India, the USA and globally. We also have a Corporate Alliance Program to bring together biopharma companies engaged in drug development. We then facilitate connections between the patient alliances and corporate alliances to help expand treatment options, alongside academic researchers, the NIH and the FDA, who provide guidelines for rare-disease research programs. This approach helps us connect the dots and expedite cross-border collaborations. I chaired the inaugural Indo US Bridging RARE Summit 2023 that was held at the George Mason University Campus in Arlington, VA in October, where keynote speakers included Peter Marks, Director of the Center for Biologics Evaluation and Research (CBER) within FDA; Vikram Karnani, Executive Vice President at Horizon Therapeutics (now Amgen); and Leimapokpam Swasticharan, Director General of Health Services, Ministry of Health and Family Welfare (MoHFW), Government of India. The Summit planning committee, comprising 11 members, honored the legends of rare disease research and cross-border collaborations, Padmashri Ishwar Chander Verma and William A. Gahl.

## **Preethi:** What are the global trends for the number of rare disease cases in recent times? And, when focusing on India, what legislative measures are being put in place to reduce the occurrence of rare diseases?

**Harsha:** In 2012, when I began my work in the rare disease field, the number of recognized rare diseases was estimated at 7000. Today, the total count stands at roughly 10,800. This means, since then, on average ∼400 new diseases have been identified annually. The Indian National Policy for Rare Diseases (NPRD) 2021 emphasizes prevention, recognizing the absence of treatments for 95% of the diseases. The policy focuses on disease prevention and genetic testing, including prenatal and premarital testing, to identify potential carriers for genetic diseases. The ability to diagnose genetic diseases has significantly improved, with the advent of next-generation sequencing (NGS) labs, making genetic testing more accessible. However, one noticeable challenge still remaining is the scarcity of genetic counsellors to interpret and guide patients on the basis of their genetic test results, which highlights the need for enhanced genetic counselling services to complement genetic testing efforts.

## **Preethi:** Why are people from low- or middle-income countries underrepresented in clinical trials?

**Harsha:** The majority of clinical trials (∼80%) take place in academic medical centers within the USA and Europe, who enjoy significant federal and private funding with a well-established regulatory ecosystem compared to the rest of the world. Usually, people living within a 50-mile radius of these academic medical centers are most likely to learn about and enrolling in these trials.

## **Preethi:** So how can we effectively address the issue of underrepresentation regarding diverse racial and ethnic populations in clinical trials?

**Harsha:** There are multiple ways to do it. Let's say, there is scope in the FDA to make diversity, equity and inclusion their top priority. This means that pharmaceutical companies proposing clinical trial protocols must include a plan to involve participants from diverse racial and ethnic backgrounds living in the USA. This approach of proportional representation can also help address concerns related to genetic variation between racial and ethnic groups. Here, we should also keep in mind that lifestyle variations may exist between, for example, Indians living in the USA versus those living in India, or other countries. Therefore, the other approach would be that the FDA includes a plan to seek the expansion of clinical trial locations through the Global Clinical Trial Network. This can be implemented by establishing investigation sites in multiple countries, which not only would allow patient representation during the clinical trial phase but also access to treatments during clinical development.

## **Preethi:** This brings me to the next question. What does it take for a country, investors and academic institutions to execute a clinical trial, and make therapies available to the patients?

**Harsha:** They say, “it takes a village to raise a child”. Generally, well-equipped academic and medical institutions are needed to execute clinical trials. Further, it involves multiple stakeholders, government regulations, guidelines to the pharma industries and national policies – like the Orphan Drug Act. In the case of cell and gene therapies, it takes special manufacturing processes, supply chain activities on a per-patient basis, high-quality clinical practices, good manufacturing practice controls, and an early research and development mindset. It also takes the patient groups to have the courage, patience and vision, to diligently work and collaborate to push the needle, and accelerate research and clinical trial processes. So, this entire ecosystem is needed in a country to have the ability to make innovative new medicines.

## **Preethi:** Despite these tremendous efforts in drug development, they can unfortunately become inaccessible to many patients due to exorbitant costs. Some parents may need to spend their entire life savings to treat their child with a rare disease.

**Harsha:** Absolutely! You see, when a drug is approved, most of them come from American or European companies. Consequently, these drugs or therapies are available at the US price, which is unaffordable in low- and middle-income countries like India. Moreover, ∼90% of people in India pay healthcare expenses out of their own pockets because they do not have insurance or their insurance providers do not cover the expenses of the orphan drugs. So, the government needs to pave the way for orphan drug companies from the USA to enter the Indian market with pricing controls that make sense within the Indian context.

[…] given the relatively small number of patients with certain rare diseases, there aren't sufficient data points within any single country to fully understand their natural history and progression. Hence, it is important to recognize each rare disease as a global public health challenge.

## **Preethi:** You also established Jeeva Clinical Trials to remotely engage rare disease patients in screening, education and participation in clinical studies. What was the motivation in establishing this platform?

**Harsha:** As a data scientist during the early part of my career, I observed that the majority of genome sequencing data, clinical trial data and patient registries primarily represented the Caucasian population.

In addition, given the relatively small number of patients with certain rare diseases, there aren't sufficient data points within any single country to fully understand their natural history and progression. Hence, it is important to recognize each rare disease as a global public health challenge.

Another challenge arises from the timelines and costs associated with bringing rare-disease therapies to the market. On average, it costs ∼2.5 billion US dollars and, typically, takes 10–12 years, from identification of a drug target/candidate to FDA review/approval. To expedite this process and enhance its efficiency, I founded Jeeva Clinical Trials, with a vision to globalize rare-disease research and clinical trials, with a digital platform. Our aim is to democratize clinical trials and the resulting innovative treatment options. Families are more likely to participate when they can engage in these activities without significant travel or logistical burdens. They are already grappling with the economic, physical and mental challenges posed by the disease itself, so if they must travel monthly to distant locations like Boston, Houston or the Bay Area, even if living within the USA, it can be incredibly challenging. Often, this logistical burden is a key reason why many patients choose not to enrol in trials. Jeeva Clinical Trials was established to simplify remote access to clinical trials for patients and to help pharmaceutical companies efficiently advance candidate drugs through the various phases of clinical trials.

Whereas IndoUSrare focuses more on awareness, research programmes and making connections between patient groups and industry, Jeeva focuses on building a software technology platform because most of the software products in clinical research are predominantly developed for English-speaking populations, with many other languages still missing. So, we are working towards making Jeeva an inclusive software platform that can be used by both diverse cultures and languages.

## **Preethi:** When discussing technology, it is compelling to be reminded of the expanding potential of AI, especially in diagnosis.

**Harsha:** Indeed! In the context of AI-based innovation, data access is crucial. Organizations with access to technologies, like facial recognition, have the potential to develop applications that can identify craniofacial features and link them to rare diseases. These applications necessitate a substantial number of photos, possibly hundreds or thousands, for effective machine-learning model training. However, it is not just about possessing these photos, we must obtain consent from the patients who contributed them for research and machine learning model training, which is an entirely different challenge.

[…] it is the researchers, who need to be thoughtful about obtaining proper informed consent. The problem arises when data have been collected without obtaining appropriate informed consent. In such cases, there is a wealth of data sitting there that cannot be used or shared –neither ethically nor legally.

## **Preethi:** Do patients typically hesitate in sharing their photographs or samples for research purposes?

**Harsha:** More than 90% of patients with rare diseases are willing to contribute and donate their samples for research. They often express, ‘Use my samples or data, do something, and help me out.’ However, it is the researchers, who need to be thoughtful about obtaining proper informed consent. The problem arises when data have been collected without obtaining appropriate informed consent. In such cases, there is a wealth of data sitting there that cannot be used or shared – neither ethically nor legally.

**Preethi:** Thanks Harsha, for this wonderful conversation!

## Conclusions

Each rare disease has unique traits, yet the challenges faced by patients and their families are strikingly similar. Frequently, patients undergo the diagnostic odyssey and have limited treatment options. These challenges are exacerbated by socio-economic inequalities, especially in lower income countries. In these scenarios, numerous hurdles persist, including limited access to adequate health insurance, clinical trials and the non-recognition of many ultra-rare diseases. Overcoming these barriers necessitates cross-border and multi-stakeholder collaborations to share knowledge, resources and guidelines for best practices. Multinational patient-advocacy groups, like IndoUSrare, are instrumental in addressing the unique challenges faced by individuals affected by rare diseases. These organisations play a vital role in raising awareness, providing support and information, advancing research and advocating for policy changes to improve the lives of those living with rare conditions. Therefore, by engaging with patient advocacy groups, researchers can bridge the gap between scientific innovation and the real-world needs of patients.
